# Maternal iodine levels and associations with offspring outcomes and growth: a prospective birth cohort study of Chinese pregnant women

**DOI:** 10.3389/fendo.2025.1714711

**Published:** 2025-12-17

**Authors:** Liangliang Huo, Tingting Zhao, Jing Wang, Xuhui Zhang, Hong Xu, Bing Zhu, Xingyi Jin, Sujuan Zhu, Qilin Sheng

**Affiliations:** 1 Hangzhou Center for Disease Control and Prevention (Hangzhou Health Supervision Institution), Hangzhou, China; 2 Zhejiang Key Laboratory of Multi-Omics in Infection and Immunity, Hangzhou, China; 3 Department of Nutrition and Food Safety, Zhejiang Province Center for Diseases Control and Prevention, Hangzhou, China

**Keywords:** birth weight, iodine, low birth weight, pregnancy, premature birth, small for gestational age

## Abstract

**Background:**

Iodine deficiency during pregnancy has been associated with various adverse outcomes; however, recent data on iodine status among women in Hangzhou, China, remain limited.

**Methods:**

Between 2019 and 2022, this birth cohort study enrolled 290 eligible pregnant women at ≤12 weeks of gestation. A standardized, self-developed questionnaire was used to survey each participant, covering demographic information, pregnancy details, dietary iodine intake, and related topics. Physical examination findings of each participant and their offspring were collected. Maternal urinary iodine concentration was measured in the first, second, and last trimesters of pregnancy. Offspring outcomes were evaluated by measuring weight and length at birth, 1 month, 6 months, and 18 months, along with the recording of incidences of spontaneous premature birth, small for gestational age (SGA), and low birth weight.

**Results:**

Higher maternal urinary iodine concentration (UIC) during the third trimester was found to be a protective factor against spontaneous premature birth and SGA. Similarly, elevated maternal UIC in the second trimester was identified as a protective factor against spontaneous premature birth. No significant association was observed between low birth weight and maternal UIC in the first, second, and last trimesters. However, offspring born to mothers with iodine insufficiency in the last trimester exhibited lower birth weight and length, as well as reduced length during follow-up. Additionally, offspring of mothers with iodine insufficiency in the first trimester showed lower long-term weight and length.

**Conclusions:**

These findings highlight the importance of enhanced monitoring of iodine status in pregnant women to mitigate related adverse outcomes.

## 1 Introduction

Iodine is an essential micronutrient required for the synthesis of thyroid hormones, such as triiodothyronine (T3) and thyroxine (T4) (1). Iodine deficiency results in reduced serum levels of these hormones, leading to iodine deficiency disorders (IDDs). Iodine deficiency poses a significant public health concern during pregnancy in both developed and developing countries (2). Pregnant women and infants are particularly vulnerable due to increased maternal iodine requirements throughout gestation, which are necessary to support thyroid hormone production for both the mother and fetus, as well as to facilitate fetal growth and development (3, 4). Studies have demonstrated that iodine deficiency during pregnancy is associated with numerous complications, including congenital abnormalities, stillbirth, spontaneous abortion, fetal growth retardation, increased infant mortality, and endemic cretinism (3, 5–7).

Universal salt iodization (USI) is recognized as the safest, most effective, economical, and convenient strategy for preventing IDDs (8). Since 1995, China has implemented USI with a mandated salt iodine concentration of 35 ± 15 mg/kg, effectively controlling IDDs among the population (9, 10). However, in Hangzhou, China, the salt iodine concentration was reduced to 25 ± 5 mg/kg in 2012, reflecting considerations of local dietary habits, iodized salt coverage, and urinary iodine concentration (UIC) (11). In recent years, reforms in China’s salt industry have increased the availability of non-iodized salt to residents (12), contributing to a gradual decline in iodized salt coverage (13, 14). Additionally, iodine nutritional status is influenced by various factors, including environmental iodine levels and residents’ dietary patterns (10). Hangzhou, located in Zhejiang Province, is characterized by relatively low natural iodine sources. Economic development has altered local dietary habits, potentially affecting the iodine status of pregnant women. The combined effect of these factors has resulted in a decrease in the median UIC among pregnant women in Hangzhou in recent years (15). This finding indicates that a subset of this population remains at risk of subclinical iodine deficiency, falling short of the iodine nutrition standards recommended by the World Health Organization (WHO) for pregnant women (16).

UIC is a widely used indicator for assessing the iodine nutritional status of pregnant women. According to the WHO, iodine deficiency during pregnancy is defined as a median UIC below 150 μg/L (16). Consequently, numerous studies evaluate iodine nutritional status and its association with adverse obstetric outcomes by measuring UIC during pregnancy. However, most of these studies rely on a single spot urine sample, which may not accurately reflect iodine status across the entire gestational period (17). Additionally, some studies focus solely on the association between urinary iodine levels during pregnancy and adverse birth outcomes—such as spontaneous premature birth, small for gestational age (SGA), and low birth weight—without considering the potential long-term impact on offspring growth and development (18).

To address these limitations, this study aimed to establish a birth cohort in Hangzhou, China, to assess the iodine nutritional status of pregnant women at various stages of pregnancy. The study further investigated the association between maternal iodine nutritional status and adverse birth outcomes, such as spontaneous premature birth, SGA, and low birth weight. Additionally, the impact of maternal iodine nutritional status on the long-term growth trend of offspring—including weight and length at birth, 1 month, 6 months, and 18 months—was evaluated.

## 2 Materials and methods

### 2.1 Study design and participants

From 2019 to 2022, a cohort study was conducted among pregnant women in Hangzhou, China. Chun’an County Maternal and Child Health Hospital serves as the primary maternal and child healthcare institution in the region, covering a substantial and representative proportion of the local pregnant population. The hospital handles a high volume of prenatal examinations and adheres to uniform, standardized protocols for prenatal care, physical examinations, and sample collection. A hospital-based consecutive sampling method was employed for participant recruitment. Sample size estimation was performed using the R package pmsampsize for generalized linear models, with premature birth as the primary outcome. Based on China’s premature birth rate of 6.9% in 2014 (19), the minimum required sample size was calculated to be 308. Accounting for a potential 10% loss to follow-up rate, the final recruitment target was determined to be 339 pregnant women. Pregnant women at ≤12 weeks of gestation who were undergoing their first prenatal examination in Hangzhou Chun’an County Maternal and Child Health Hospital were enrolled. A birth cohort was established to investigate and follow up on pregnant women and their offspring. Women who were (1) aged 18–45 years and able to communicate effectively; (2) had resided in the local area for at least 1 year with plans to remain for the next 3 years; (3) pregnant with a singleton pregnancy at ≤12 weeks of gestation; and (4) willing to complete regular questionnaire investigations and physical examinations as required by the research and provide informed consent were included in the study. Conversely, women who (1) had a family history of thyroid disease; (2) developed chronic diseases such as diabetes, hypertension, and thyroid disease; and (3) received an injection of iodine contrast medium (used in X-ray, computed tomography, or angiography) within the past year were excluded. All participants received detailed physical examination reports and routine prenatal check-up results. Furthermore, clinical experts from our research team provided brief health consultations based on these test results, thereby offering direct health benefits to the participants. We explicitly assured all participants that their decision to participate in or withdraw from the study would not affect the quality or cost of their routine medical care.

### 2.2 Questionnaire investigation and physical examination

A face-to-face questionnaire was administered to each participant by an obstetrician who had received standardized training. A standardized, self-developed questionnaire was used to survey each participant. The questionnaire, having been pretested and refined, covered demographic information, pregnancy details, dietary iodine intake, and related topics. All participants recruited in this study received routine antenatal care. Data regarding antenatal examinations and delivery were obtained from the hospital’s medical record system. In addition, physical examinations of both pregnant women and their infants were performed by the attending physicians. These examinations included measurements of pregnant women’s weight and height and infant’s weight and length at birth, 1 month, 6 months, and 18 months.

### 2.3 Sampling and testing methods and criteria for evaluating urinary iodine concentration

During the first trimester (≤12 weeks), second trimester (12–27 weeks), and last trimester (≥28 weeks), at least two random urine samples of 20 ml each were collected from pregnant women. These samples were stored at −20 °C without the addition of preservatives or chemical reagents until analysis. The UIC was determined by the As^3+^–Ce^4+^ Catalytic Spectrophotometry, using a 723N visible spectrophotometer (Shanghai Spectrum Instruments Co., Ltd., China) at 420 nm. All laboratories conducting iodine testing had participated in external quality control assessments organized by the Chinese Center for Disease Control and Prevention. The average of two or more random UIC measurements was used to represent each pregnant women’s iodine status in the first, second, and last trimesters. Iodine nutritional status was evaluated according to the WHO’s 2007 guidelines for IDD in pregnant women. Considering the environmental iodine deficiency in Hangzhou and the low urinary iodine levels observed among the participants, UIC values were stratified into three categories: <100 μg/L, 100–149 μg/L, and ≥150 μg/L.

### 2.4 Evaluation method and outcome criteria

The outcomes assessed for offspring in this study included infant weight and length at birth, 1 month, 6 months, and 18 months, as well as spontaneous premature birth, SGA, and low birth weight. Spontaneous premature birth was defined as delivery before 37 weeks of gestation. SGA was defined as newborns with a birth weight or length below the 10th percentile of the normal reference values for the corresponding gestational age, based on the growth reference curves for neonatal weight and length developed by the Capital Institute of Pediatrics in China (20). Low birth weight was defined as a birth weight of less than 2.5 kg.

### 2.5 Statistical analysis

EpiData software (version 3.1) was used to establish a unified database, with questionnaires uniformly numbered, checked, and subjected to double data entry. Data analysis was performed using R software (version 3.6.0). UIC was expressed as the median with interquartile range, whereas infant weight and length were expressed as the mean ± standard deviation (SD). The Kruskal-Wallis H tests were performed to compare maternal UIC across groups with different characteristics. Multiple linear regression analysis was conducted to identify factors influencing UIC during pregnancy. ANOVA was used to compare infant weight and length at various ages according to maternal urinary iodine levels. A generalized linear model was applied to assess the potential effects of maternal urinary iodine levels on offspring birth outcomes (including spontaneous premature birth, SGA, and low birth weight). Variable selection was performed using the optimal subset method, and adjustments were made for confounding factors (including age, education, average annual household income, BMI at first visit, smoking or passive smoking status, alcohol consumption, type of salt consumed, frequency of iodine-rich food intake, use of iodine-containing supplements, neonatal sex, and mode of delivery). Generalized estimating equations (GEEs) were used to explore long-term trends in infant weight and length in relation to maternal urinary iodine levels, with adjustments for birth weight or length and the same confounders mentioned above. A p-value of <0.05 was considered significant, and all reported results included robust 95% confidence intervals (CIs). The research data are available from the corresponding author upon reasonable request.

### 2.6 Ethics statement

This study was approved by Medical Ethics Committee of Zhejiang Province Center for Disease Control and Prevention (Approval No: T-043-R). Informed consent was obtained from all participants included in the study.

## 3 Results

### 3.1 Demographic characteristics of all participants

**Figure 1:**
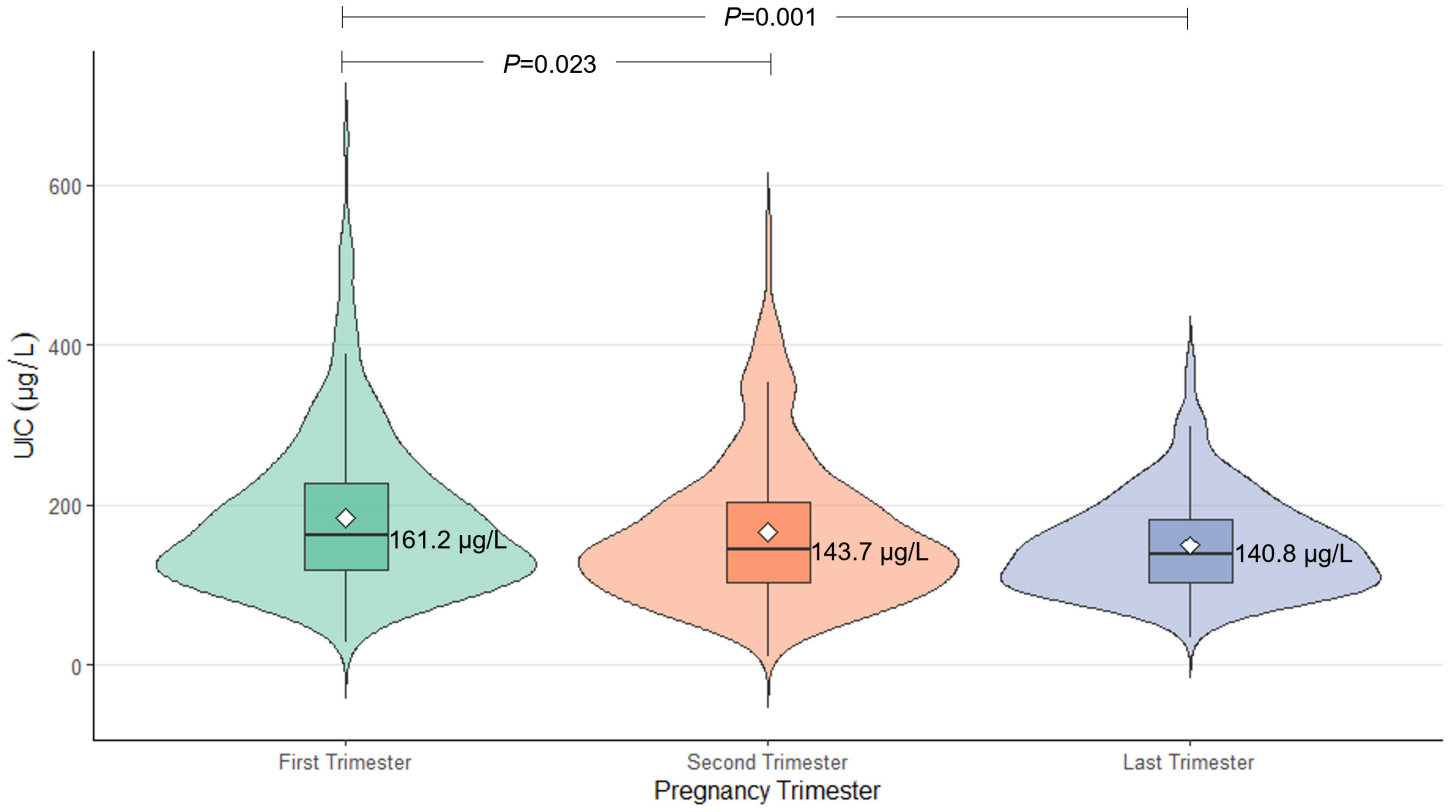
Distribution of UIC in pregnant women across different trimesters. A p-value of <0.05 was considered statistically significant.

Between February 2019 and February 2022, we recruited pregnant women who were undergoing their first prenatal examination in Hangzhou Chun’an County Maternal and Child Health Hospital. A total of 344 pregnant women who met the inclusion and exclusion criteria agreed to participate in the investigation. During the subsequent follow-up, 290 pregnant women and their offspring were ultimately enrolled in this investigation after excluding participants who experienced miscarriage or induced abortion (n=29), stillbirth (n=1), or drop out of the study (n=24). All participants completed a face-to-face questionnaire and provided urine samples in the first, second, and last trimesters. Follow-up was completed for all 290 participants (100.00%) until delivery, for 285 participants (98.28%) until the infants reached 1 month old, 277 participants (95.52%) until the infants reached 6 months old, and for 200 participants (68.97%) until the infants reached 18 months old. The mean maternal age was 30.1 years (SD: 4.2 years). The average maternal body mass index (BMI) at the first visit was 21.6 kg/m^2^. The median UIC in the first trimester was 161.2 μg/L, meeting the recommended level set by the WHO. However, the median UIC values in the second and last trimesters were 143.7 μg/L and 140.8 μg/L, respectively, falling below the recommended threshold. Furthermore, the median UIC in the first trimester was significantly higher than those in the second and last trimesters (P = 0.023 and P = 0.001, respectively). These results are presented in **Figure 1**.

When comparing the median UIC in pregnant women with different demographic characteristics across trimesters, the study found that during the first trimester, women aged 35 years or older exhibited significantly lower median UIC (P = 0.035), whereas those who smoked or were exposed to secondhand smoke demonstrated higher median UIC (P = 0.003). In the second trimester, alcohol consumption was associated with a lower median UIC (P = 0.044). With regard to birth outcomes, spontaneous premature birth, SGA, and low birth weight accounted for 5.5%, 6.6%, and 1.7%, respectively. Mothers of preterm infants had significantly lower median UIC in the second trimester (P = 0.026), whereas mothers of SGA and low birth weight infants exhibited lower median UIC in the third trimester (P = 0.025 and P = 0.020, respectively). Other demographic characteristics did not show a significant association with low median UIC. These findings are provided in **Table 1**. Additionally, pregnant women who consumed iodized salt had higher urinary iodine levels compared with those who did not, as shown in **Table 2**.

**Table 1:** Comparison of median UIC in pregnant women with different characteristics across trimesters.

Variable	*N* (%)		UIC [*M* (*P*_25_, *P*_75)_]					
			First trimester	P	Second trimester	P	Last trimester	P
Age								
18–34 years old	267	92.1	163.7 (120.5–229.6)	0.035*	143.4 (100.8–211.3)	0.668	144.9 (107.7–185.3)	0.893
≥35 years old	23	7.9	126.8 (109.0–183.1)		144.1 (109.4–182.7)		126.8 (92.8–162.1)	
Education								
Junior middle school and below	31	10.7	156.1 (116.6–196.4)	0.528	148.3 (108.9–193.6)	0.314	140.1 (118.2–179.4)	0.550
Senior middle school	97	33.4	170.6 (121.5–223.6)		152.8 (115.8–211.3)		132.0 (103.8–176.0)	
College degree or above	162	55.9	159.0 (114.5–235.3)		137.8 (95.4–196.3)		144.8 (106.3–186.8)	
Average annual household income								
≤29,999￥	17	5.9	154.6 (110.8–189.7)	0.898	184.8 (97.9–197.8)	0.601	136.9 (108.6–202.8)	0.949
30,000–59,999￥	47	16.2	162.1 (117.5–206.3)		155.1 (113.7–220.0)		149.7 (107.6–182.6)	
60,000–99,999￥	55	19.0	160.0 (123.6–228.3)		133.4 (103.4–210.9)		134.5 (107.9–178.7)	
≥100,000￥	171	59.0	163.7 (116.8–235.3)		143.1 (100.0–196.9)		140.7 (103.9–182.0)	
BMI at first visit								
<18.0	14	4.8	131.2 (97.01–183.8)	0.151	147.0 (129.5–213.4)	0.301	112.8 (104.3–161.2)	0.742
18.0–23.9	220	75.9	169.2 (121.3–233.7)		148.3 (103.9–210.7)		140.4 (106.0–187.1)	
≥24	56	19.3	150.8 (113.8–209.4)		134.5 (97.9–179.4)		146.3 (108.7–176.5)	
Smoking or passive smoking								
Yes	34	11.7	214.8 (159.3–254.7)	0.003*	145.7 (107.3–199.0)	0.724	141.7 (106.4–184.8)	0.498
No	256	88.3	154.6 (113.3–215.4)		143.7 (102.4–203.4)		135.4 (97.6–180.0)	
Alcohol consumption								
Yes	7	2.4	214.2 (164.5–257.7)	0.222	99.9 (71.4–127.0)	0.044*	136.4 (116.9–196.5)	0.875
No	283	97.6	160.0 (117.7–224.8)		144.9 (104.8–204.3)		140.9 (105.6–184.2)	
Type of salt consumed								
Iodized salt	197	67.9	156.1 (111.0–229.5)	0.114	140.2 (100.8–197.7)	0.618	137.7 (103.8–180.7)	0.064
Non-iodized salt	6	2.1	122.4 (102.8–153.2)		125.8 (97.6–185.9)		104.2 (94.4–144.0)	
Both	26	9.0	145.7 (122.2–213.9)		150.9 (92.9–218.0)		140.8 (104.3–175.3)	
Unknown	61	21.0	180.5 (132.5–246.6)		155.2 (111.2–212.5)		163.1 (119.0–202.2)	
Frequency of iodine-rich food intake								
Less	97	33.1	175.0 (109.8–216.9)	0.410	148.4 (103.6–203.0)	0.144	152.2 (105.4–181.5)	0.815
Medium	178	61.4	187.7 (124.2–227.0)		148.1 (105.6–211.3)		136.4 (106.1–186.0)	
More	15	5.2	192.8 (120.6–217.8)		115.2 (85.4–134.6)		133.7 (111.7–185.3)	
Use of iodine-containing supplements								
Yes	48	16.6	168.2 (116.7–215.1)	0.995	153.7 (112.3–198.2)	0.679	159.0 (128.3–188.4)	0.057
No	242	83.4	160.1 (118.2–227.0)		143.0 (102.4–204.6)		137.9 (104.2–180.7)	
Neonatal sex								
Male	162	55.9	169.2 (120.0–232.2)	0.379	142.0 (103.9–218.7)	0.924	136.7 (103.5–184.6)	0.377
Female	128	44.1	150.9 (116.0–224.2)		149.1 (102.3–189.7)		147.4 (108.9–181.2)	
Mode of delivery								
Natural delivery	161	55.5	173.1 (125.8–217.8)	0.067	149.4 (106.5–210.5)	0.344	141.6 (106.2–186.4)	0.722
Cesarean	129	44.5	149.9 (103.5–233.2)		141.2 (97.9–190.8)		137.7 (105.0–180.3)	
Spontaneous premature birth								
Yes	16	5.5	177.1 (118.8–251.3)	0.824	124.1 (96.7–143.0)	0.026*	110.2 (80.2–164.4)	0.081
No	274	94.5	160.6 (117.9–225.4)		148.2 (104.0–209.3)		143.6 (106.4–184.3)	
SGA								
Yes	19	6.6	141.2 (92.6–192.0)	0.193	141.0 (115.6–205.8)	0.785	108.6 (87.6–162.2)	0.025*
No	271	93.4	162.1 (118.3–228.2)		144.9 (102.7–203.1)		144.7 (106.4–185.6)	
Low birth weight								
Yes	5	1.7	126.0 (109.0–253.0)	0.700	143.4 (142.9–151.2)	0.700	72.3 (68.9–73.6)	0.020*
No	285	98.3	161.0 (118.0–226.0)		144.0 (103.0–204.0)		142.0 (106.0–184.0)	

**Table 2:** Analysis of factors influencing UIC during pregnancy.

Variable	*β*	*S.E.*	*t*	P
Age				
18–34 years old	Ref			
≥35 years old	−13.5230	10.2410	−1.320	0.1878
Education				
Junior middle school and below	Ref			
Senior middle school	−6.3025	9.7220	−0.648	0.5173
College degree or above	−6.9263	9.5274	−0.727	0.4679
Average annual household income				
≤29,999￥	Ref			
30,000–59,999￥	−4.3813	13.1867	−0.332	0.7400
60,000–99,999￥	−14.3106	13.0189	−1.099	0.2726
≥100,000￥	−8.5940	12.2247	−0.703	0.4826
BMI at first visit	−0.2338	0.9399	−0.249	0.8038
Smoking or passive smoking				
No	Ref			
Yes	11.0937	9.2393	1.201	0.2309
Alcohol consumption				
No	Ref			
Yes	7.5682	18.8223	0.402	0.6879
Salt consumption type				
Other				
Iodized salt	5.5302	2.3265	2.377	0.0181^*^
Frequency of iodine-rich food consumption				
Less				
Medium	1.6676	5.8843	0.283	0.7771
More	−22.3944	13.2805	−1.686	0.0929
Use of iodine-containing supplements				
No				
Yes	−10.4492	7.5480	−1.384	0.1674

* P<0.05.

### 3.2 Differences in offspring weight and length at various ages according to maternal UIC

Pregnant women in this study were divided into three groups based on their UIC. Group 1 comprised participants with a UIC of <100 μg/L, group 2 comprised those with a UIC of 100–149 μg/L, and group 3 comprised those with a UIC of ≥150 μg/L. Offspring weight and length of various ages were compared across these maternal UIC groups. The findings revealed that lower maternal UIC during the first trimester was significantly associated with reduced offspring weight and length at 18 months of age (P = 0.042 and P = 0.013). Moreover, lower maternal UIC in the third trimester showed a significant correlation with lower birth weight (P = 0.001). Maternal UIC levels were also significantly associated with infant length at birth, 1 month, 6 months, and 18 months (P<0.001, P = 0.005, P<0.001, and P = 0.011). These findings are summarized in **Table 3**.

**Table 3:** Comparison of offspring weight and length at different ages according to maternal UIC.

Trimester	Measurement time	Variable	Group 1^#^	Group 2^#^	Group 3^#^	P
First trimester	At birth	*N*	46	85	159	
		Weight ( $\bar{\text{x}}\pm s$)	3.28 ± 0.46	3.31 ± 0.46	3.28 ± 0.46	0.896
		Length ( $\bar{\text{x}}\pm s$)	50.0 ± 1.4	50.1 ± 1.5	50.0 ± 1.4	0.974
	At 1 month	*N*	44	84	157	
		Weight ( $\bar{\text{x}}\pm s$)	4.41 ± 0.65	4.35 ± 0.55	4.36 ± 0.50	0.848
		Length ( $\bar{\text{x}}\pm s$)	54.7 ± 2.2	54.5 ± 2.0	54.8 ± 1.9	0.709
	At 6 months	*N*	43	81	153	
		Weight ( $\bar{\text{x}}\pm s$)	7.98 ± 0.85	8.11 ± 0.89	8.07 ± 0.96	0.769
		Length ( $\bar{\text{x}}\pm s$)	67.7 ± 2.1	67.7 ± 2.2	68.1 ± 2.2	0.264
	At 18 months	*N*	29	50	121	
		Weight ( $\bar{\text{x}}\pm s$)	10.37 ± 0.98	10.75 ± 1.02	10.91 ± 1.06	0.042*
		Length ( $\bar{\text{x}}\pm s$)	81.5 ± 3.1	81.7 ± 2.5	82.7 ± 2.4	0.013*
Second trimester	At birth	*N*	67	86	137	
		Weight ( $\bar{\text{x}}\pm s$)	3.30 ± 0.51	3.31 ± 0.48	3.28 ± 0.42	0.888
		Length ( $\bar{\text{x}}\pm s$)	50.0 ± 1.6	50.1 ± 1.4	50.0 ± 1.4	0.751
	At 1 month	*N*	65	86	134	
		Weight ( $\bar{\text{x}}\pm s$)	4.39 ± 0.54	4.34 ± 0.57	4.38 ± 0.52	0.821
		Length ( $\bar{\text{x}}\pm s$)	54.6 ± 2.2	54.7 ± 1.9	54.7 ± 1.9	0.837
	At 6 months	*N*	62	83	132	
		Weight ( $\bar{\text{x}}\pm s$)	8.18 ± 1.08	8.12 ± 0.90	8.00 ± 0.86	0.402
		Length ( $\bar{\text{x}}\pm s$)	67.9 ± 2.5	68.2 ± 1.9	67.7 ± 2.1	0.332
	At 18 months	*N*	48	48	104	
		Weight ( $\bar{\text{x}}\pm s$)	10.80 ± 1.09	10.77 ± 0.97	10.79 ± 1.08	0.989
		Length ( $\bar{\text{x}}\pm s$)	82.2 ± 2.9	82.4 ± 2.6	82.3 ± 2.4	0.921
Last trimester	At birth	*N*	58	103	129	
		Weight ( $\bar{\text{x}}\pm s$)	3.10 ± 0.50	3.34 ± 0.43	3.33 ± 0.44	0.001*
		Length ( $\bar{\text{x}}\pm s$)	49.4 ± 1.8	50.3 ± 1.0	50.1 ± 1.4	<0.001*
	At 1 month	*N*	56	101	128	
		Weight ( $\bar{\text{x}}\pm s$)	4.23 ± 0.55	4.40 ± 0.56	4.40 ± 0.49	0.0999
		Length ( $\bar{\text{x}}\pm s$)	53.9 ± 1.9	54.9 ± 2.0	54.8 ± 1.9	0.005*
	At 6 months	*N*	53	98	126	
		Weight ( $\bar{\text{x}}\pm s$)	7.86 ± 0.72	8.11 ± 0.10	8.12 ± 0.92	0.197
		Length ( $\bar{\text{x}}\pm s$)	66.9 ± 1.9	68.2 ± 2.3	68.1 ± 206	<0.001*
	At 18 months	*N*	32	62	106	
		Weight ( $\bar{\text{x}}\pm s$)	10.44 ± 0.82	10.90 ± 1.14	10.83 ± 1.05	0.117
		Length ( $\bar{\text{x}}\pm s$)	81.0 ± 2.4	82.6 ± 2.8	82.5 ± 2.4	0.011*

### 3.3 Effects of different maternal UIC on birth outcomes in offspring

**Figure 2:**
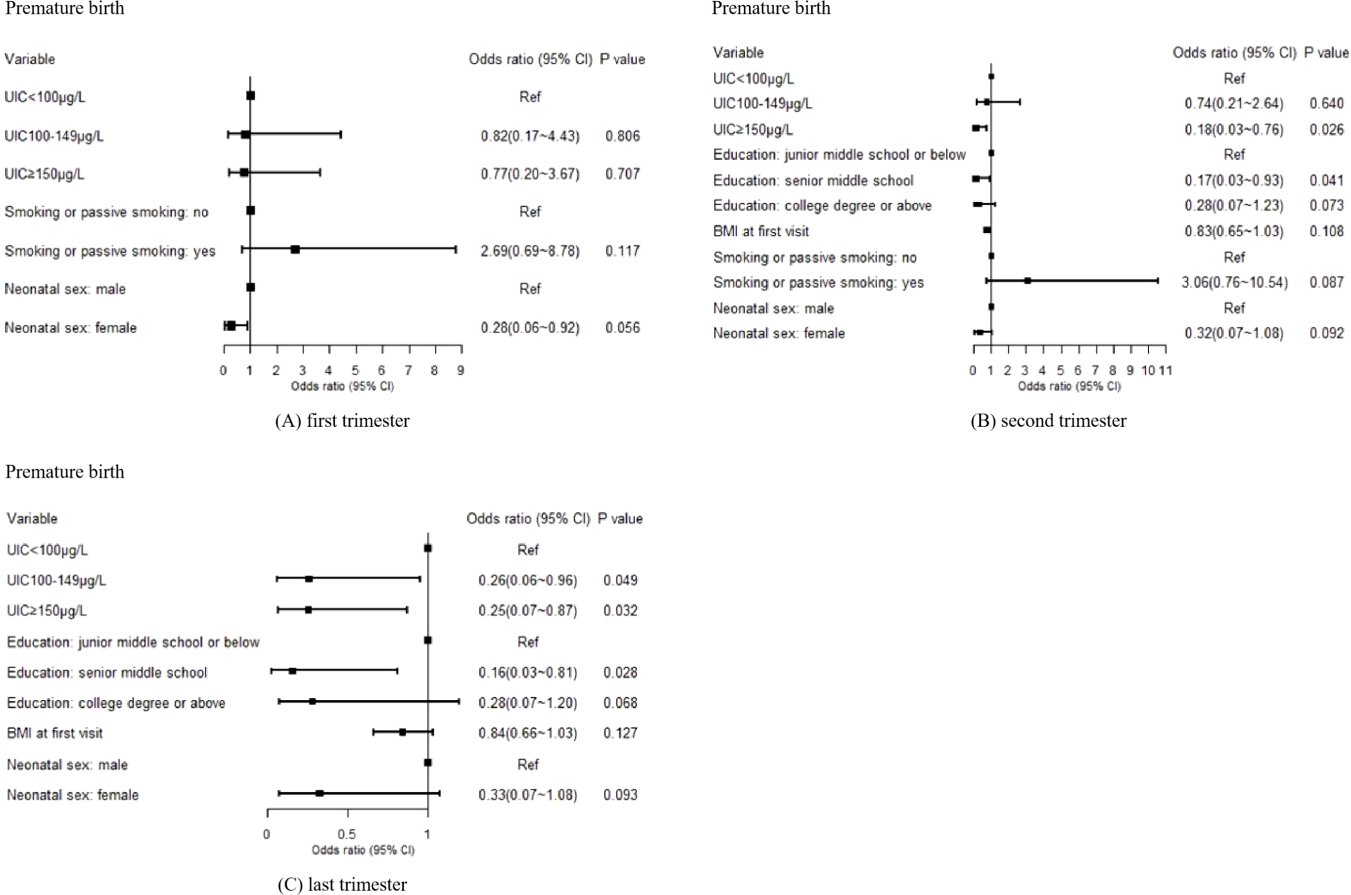
Effects of maternal UIC on the occurrence of premature birth in offspring: **(A)** first trimester, **(B)** second trimester, and **(C)** third trimester. A p-value of <0.05 was considered statistically significant.

Using spontaneous premature birth, SGA, and low birth weight as binary birth outcomes, generalized linear models were employed for analysis. After conducting variable selection and adjusting for confounding factors, a significant correlation was observed between the occurrence of spontaneous premature birth and maternal UIC in the second and third trimesters (P<0.05). Higher maternal UIC was identified as a protective factor against spontaneous premature birth. In the second trimester, the likelihood of spontaneous premature birth in the UIC≥150μg/L group was 0.18 (0.03–0.76) times lower than that in the UIC<100μg/L group. In the last trimester, the probability of spontaneous premature birth was 0.26 (0.06–0.96) times lower in the UIC100–149μg/L group and 0.25 (0.07–0.87) times lower in the UIC≥150μg/L group compared with the UIC < 100 μg/L group. However, no significant difference was found between maternal UIC in the first trimester and the occurrence of spontaneous premature birth (P>0.05). Detailed results are presented in **Figure 2**.

**Figure 3:**
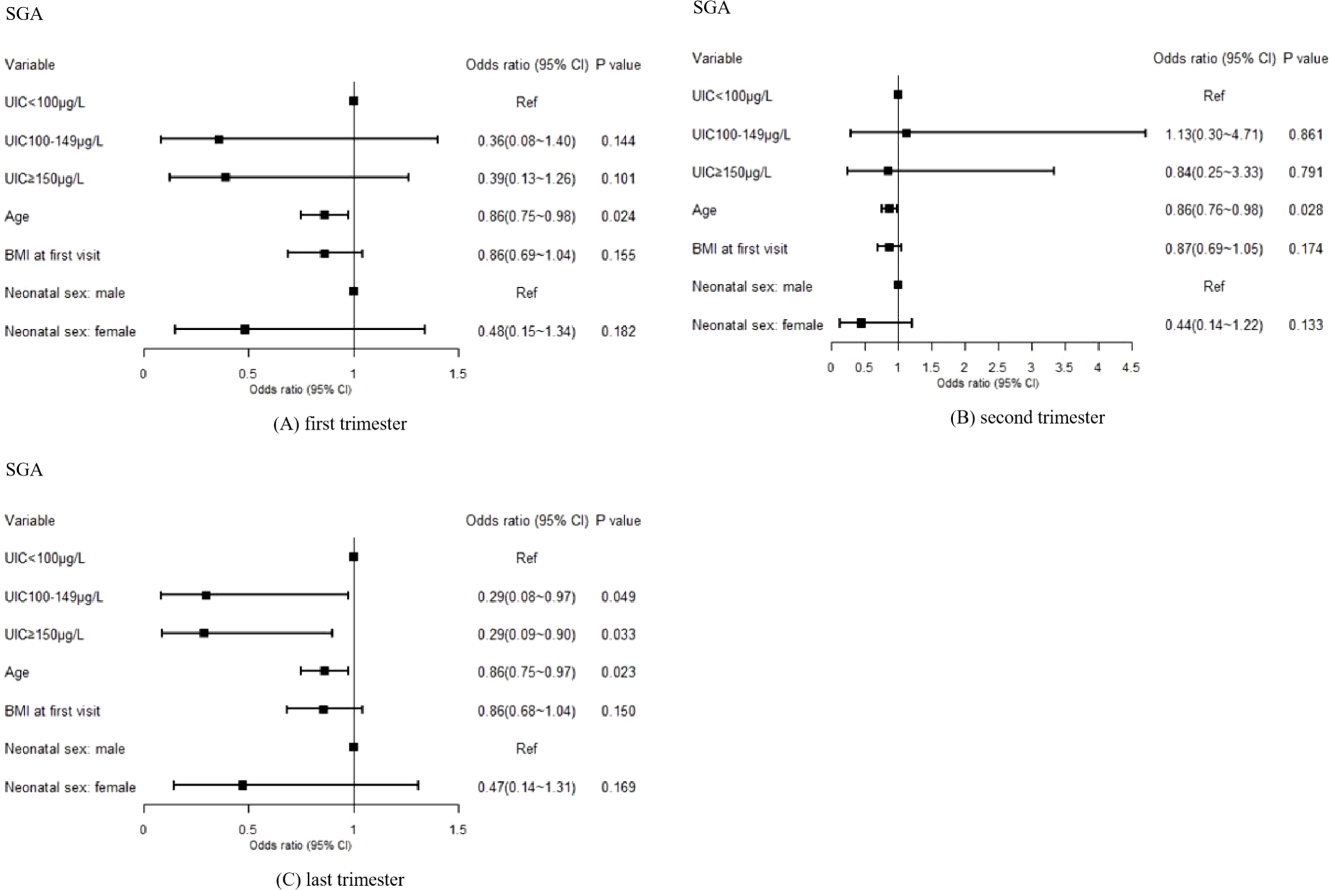
Effects of maternal UIC on the occurrence of SGA in offspring: **(A)** first trimester, **(B)** second trimester, and **(C)** third trimester. A p-value of <0.05 was considered statistically significant.

For SGA, the analysis revealed a significant association between maternal UIC in the last trimester and the risk of SGA (P<0.05). Higher maternal UIC was identified as a protective factor against SGA. In the last trimester, the risk of SGA was 0.29 (0.08–0.97) times lower in the UIC100–149μg/L group and 0.29 (0.09–0.90) times lower in the UIC≥150μg/L group compared with the UIC<100μg/L group. However, no significant association was found between maternal UIC in the first and second trimesters and the occurrence of SGA (P>0.05). Detailed results are presented in **Figure 3**.

**Figure 4:**
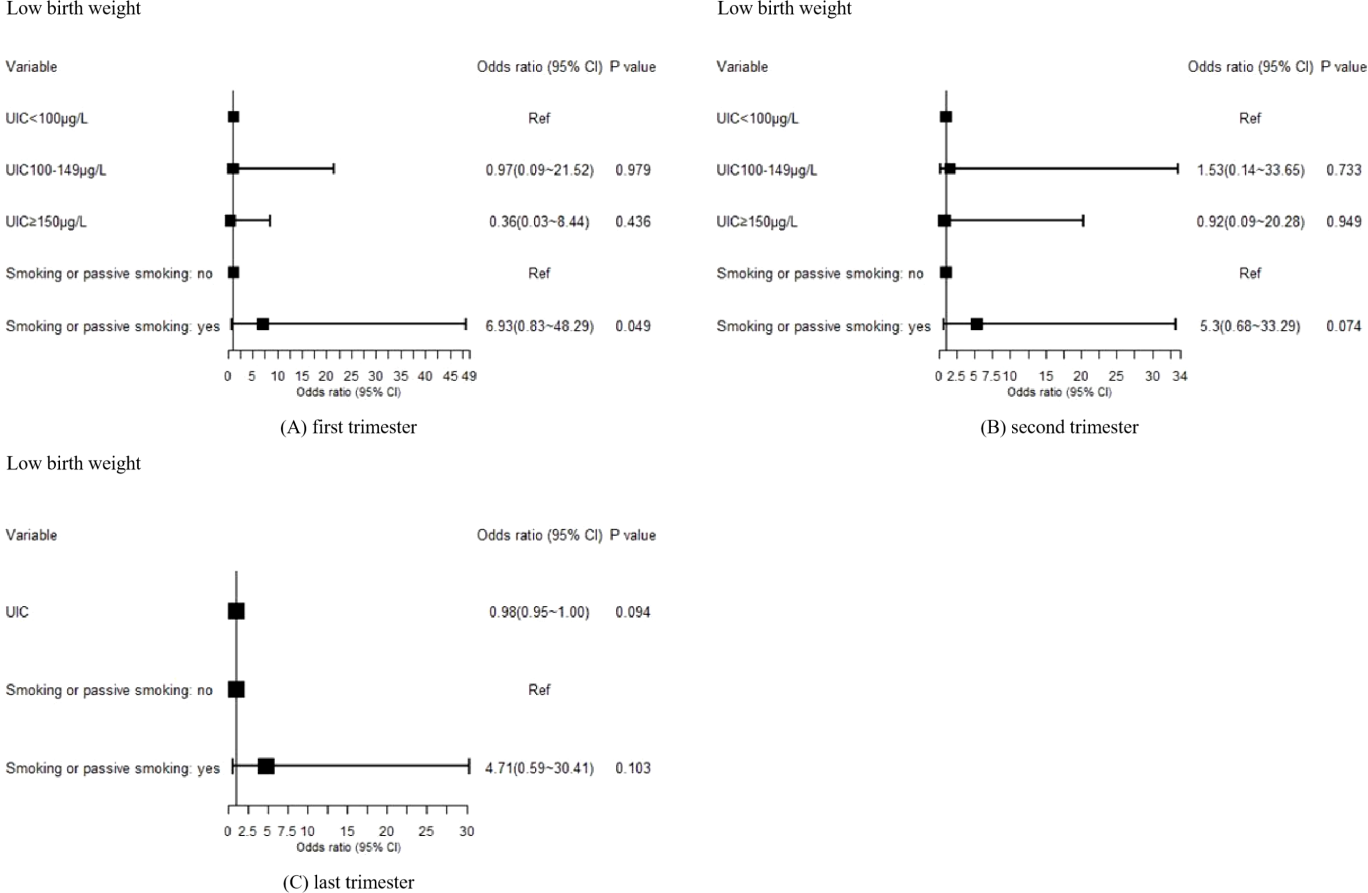
Effects of maternal UIC on the occurrence of low birth weight in offspring: **(A)** first trimester, **(B)** second trimester, and **(C)** third trimester. In the last trimester, categorical UIC variables perfectly predicted the outcome; therefore, UIC was analyzed as a continuous variable. A p-value of <0.05 was considered statistically significant.

In the analysis of low birth weight as a binary birth outcome, no significant association was found between maternal UIC in the first, second, and last trimesters and the occurrence of low birth weight (P>0.05). Detailed results are presented in **Figure 4**.

### 3.4 Trends in offspring weight and length according to maternal UIC

The present study evaluated the trends in offspring weight and length based on maternal UIC. To analyze the data, the AR1 correlation matrix was used in constructing GEE models. The findings revealed significant differences in offspring weight and length at different time points, after adjusting for birth weight and birth length in all GEE models (P<0.001). An interaction was observed between infant weight at 18 months of age and maternal UIC in the first trimester (P = 0.017). No interaction was identified between time and maternal UIC in the first trimester. Nonetheless, offspring length increased by 0.3697 (P = 0.035) in the UIC≥150μg/L group compared with the UIC<100μg/L group. Detailed results are presented in **Table 4**. In the third trimester, no interaction was found between time and maternal UIC, and offspring weight and length did not differ significantly among the maternal UIC groups (P>0.05). These results are presented in **Table 5**. Similarly, no interaction was found between time and maternal UIC in the third trimester. With regard to the main effect of maternal UIC in the last trimester, offspring length increased by 0.5062 (P = 0.0047) in the UIC100–149μg/L group compared with the UIC<100μg/L group. However, no significant difference was observed in offspring weight between these two groups (P>0.05). Compared with the UIC<100μg/L group, offspring length increased by 0.4533 (P = 0.0045) in the UIC≥150μg/L group, whereas no significant difference was detected in offspring weight between the two groups (P>0.05). These outcomes are summarized in **Table 6**.

**Table 4:** Trends in offspring weight and length according to maternal UIC in the first trimester^1^.

Weight	β	SE	Wald x^2^	P	Length	β	SE	Wald x^2^	P
(Intercept)	−0.18958	0.32330	0.34	0.558	(Intercept)	6.1063	2.8285	4.66	0.031*
Birth weight	0.89272	0.05334	280.14	<0.001*	Birth length	0.8523	0.0564	228.59	<0.001*
Time 0^2^	Ref				Time 0^2^	Ref			
Time 1^2^	1.14721	0.06748	289.03	<0.001*	Time 1^2^	4.6452	0.0870	2849.72	<0.001*
Time 2^2^	4.73816	0.09560	2456.59	<0.001*	Time 2^2^	17.9261	0.1126	25349.16	<0.001*
Time 3^2^	7.19950	0.13742	2744.67	<0.001*	Time 3^2^	32.2011	0.1572	41986.61	<0.001*
Group 1^3^	Ref				Group 1^3^	Ref			
Group 2^3^	0.04037	0.02923	1.91	0.167	Group 2^3^	0.1255	0.1883	0.44	0.505
Group 3^3^	0.01049	0.03013	0.12	0.728	Group 3^3^	0.3697	0.1754	4.44	0.035*
Time 0: Group 2	Ref				—	—	—	—	—
Time 1: Group 2	−0.09849	0.08124	1.47	0.225	—	—	—	—	—
Time 2: Group 2	0.06822	0.12648	0.29	0.590	—	—	—	—	—
Time 3: Group 2	0.27009	0.18776	2.07	0.150	—	—	—	—	—
Time 0: Group 3	Ref				—	—	—	—	—
Time 1: Group 3	−0.06769	0.07322	0.85	0.355	—	—	—	—	—
Time 2: Group 3	0.06004	0.11942	0.25	0.615	—	—	—	—	—
Time 3: Group 3	0.38384	0.16131	5.66	0.017*	—	—	—	—	—

^1^Confounding factors were adjusted, including age, educational background, average annual household income, BMI at first visit, smoking or passive smoking, alcohol consumption, type of salt consumed, frequency of iodine-rich food intake, use of iodine-containing supplements, neonatal sex, mode of delivery.

^2^Time 0, at birth; Time 1, at 1 month; Time 2, at 6 months; Time 3, at 18 months.

^3^Group 1, UIC<100μg/L; Group 2, UIC100–149μg/L; Group 3, UIC≥150μg/L.

* P<0.05.

**Table 5:** Trends in infant weight and length according to maternal UIC in the second trimester^1^.

Weight	β	SE	Wald x^2^	P	Length	β	SE	Wald x^2^	P
(Intercept)	−0.194	0.0337	0.33	0.564	(Intercept)	6.30239	2.87078	4.82	0.028*
Birth weight	0.896	0.0536	279.6	<0.001*	Birth length	0.85224	0.05645	227.93	<0.001*
Time 0^2^	Ref				Time 0^2^	Ref			
Time 1^2^	1.08	0.0232	2171.32	<0.001*	Time 1^2^	4.64559	0.08707	2846.92	<0.001*
Time 2^2^	4.79	0.0484	9781.68	<0.001*	Time 2^2^	17.9268	0.11276	25273.57	<0.001*
Time 3^2^	7.50	0.0635	13944.71	<0.001*	Time 3^2^	32.20961	0.15726	41952.1	<0.001*
Group 1^3^	Ref				Group 1^3^	Ref			
Group 2^3^	−0.0502	0.0647	0.6	0.438	Group 2^3^	0.00984	0.1796	0	0.956
Group 3^3^	−0.0476	0.0628	0.57	0.449	Group 3^3^	−0.01796	0.17233	0.01	0.917

^1^Confounding factors were adjusted, including age, educational background, average annual household income, BMI at first visit, smoking or passive smoking, alcohol consumption, type of salt consumed, frequency of iodine-rich food intake, use of iodine-containing supplements, neonatal sex, mode of delivery.

^2^Time 0, at birth; Time 1, at 1 month; Time 2, at 6 months; Time 3, at 18 months.

^3^Group 1, UIC<100μg/L; Group 2, UIC100–149μg/L; Group 3, UIC≥150μg/L.

* P<0.05.

**Table 6:** Trends in infant weight and length according to maternal UIC in the third trimester^1^.

Weight	β	SE	Wald x^2^	P	Length	β	SE	Wald x^2^	P
(Intercept)	−0.25835	0.32537	0.63	0.427	(Intercept)	7.3999	2.9443	6.32	0.0120*
Birth weight	0.88224	0.05511	256.28	<0.001*	Birth length	0.8202	0.0584	196.99	<0.001*
Time 0^2^					Time 0^2^				
Time 1^2^	1.0809	0.02321	2169.03	<0.001*	Time 1^2^	4.6451	0.087	2851.27	<0.001*
Time 2^2^	4.7909	0.04836	9814.21	<0.001*	Time 2^2^	17.9233	0.1126	25354.28	<0.001*
Time 3^2^	7.498	0.06351	13936.8	<0.001*	Time 3^2^	32.1997	0.1575	41777.17	<0.001*
Group 1^3^					Group 1^3^				
Group 2^3^	0.08343	0.06411	1.69	0.193	Group 2^3^	0.5062	0.1789	8.01	0.0047*
Group 3^3^	0.04602	0.06008	0.59	0.444	Group 3^3^	0.4533	0.1596	8.07	0.0045*

^1^Confounding factors were adjusted, including age, educational background, average annual household income, BMI at first visit, smoking or passive smoking, alcohol consumption, type of salt consumed, frequency of iodine-rich food intake, use of iodine-containing supplements, neonatal sex, mode of delivery.

^2^Time 0, at birth; Time 1, at 1 month; Time 2, at 6 months; Time 3, at 18 months.

^3^Group 1, UIC<100μg/L; Group 2, UIC100–149μg/L; Group 3, UIC≥150μg/L.

* P<0.05.

## 4 Discussion

Iodine deficiency is a prevalent micronutrient deficiency among vulnerable populations, particularly pregnant women and infants. Even subclinical iodine deficiency during pregnancy can adversely affect fetal growth and development, increasing the risk of unfavorable obstetric outcomes (2). Although IDDs were successfully eliminated among residents of Hangzhou in 2011, a proportion of pregnant women has continued to be at risk of subclinical iodine deficiency in recent years. Therefore, the present study established a birth cohort to investigate the impact of iodine deficiency during pregnancy on adverse birth outcomes and infant growth among pregnant women in Hangzhou.

Our findings revealed that following the adjustment in salt iodization strategy, pregnant women in Hangzhou exhibited mild iodine deficiency, particularly in the second and third trimesters. Previous studies have shown that even mild iodine deficiency in the mother may have long-term adverse effects on the growth and cognitive development of the offspring (21–23). Furthermore, iodine deficiency worsened as pregnancy progressed, a pattern consistent with trends observed in other coastal regions of China (15, 24–27). The underlying causes of this progressive decline in iodine status remain incompletely understood. Some studies have suggested that gestational edema may lead to a gradual reduction in the consumption of iodized salt, thereby leading to decreased UIC and warranting further investigation (15). These findings indicate that even in areas where IDDs have been declared eliminated, pregnant women continue to face a potential risk of subclinical iodine deficiency. This persistent threat underscores the critical need for sustained public health vigilance and suggests that current IDD prevention strategies may require optimization to meet the specific needs of pregnant women. We therefore recommend the implementation of continuous iodine status monitoring and targeted nutritional education for expectant mothers and their families. Furthermore, iodine fortification strategies should be promptly refined based on surveillance results.

Our study identified a higher likelihood of adverse pregnancy outcomes, such as spontaneous premature birth and SGA, among pregnant women with iodine insufficiency during the last trimester. Maternal iodine insufficiency in the second trimester was also linked to a higher risk of spontaneous premature birth, although no significant difference was observed in the occurrence of SGA. No associations were identified between maternal iodine nutritional status in the first trimester and the incidence of spontaneous premature birth or SGA. A study conducted in Spain highlighted that low UIC in the last trimester increased the risk of delivering SGA newborns (28). Similarly, a meta-analysis demonstrated that infants born to mothers with UIC levels above 150 μg/L had a lower risk of SGA, corroborating our results (5). Research in Thailand also identified iodine deficiency as an independent risk factor for premature birth, further supporting our findings (29). However, a meta-analysis cautioned against overinterpreting the association between iodine status and premature birth due to potential bias concerns (5). Although our univariate analysis indicated a relationship between urinary iodine levels in the last trimester and low birth weight, this association did not remain significant after adjusting for confounding factors. This outcome is consistent with the conclusions of a systematic literature review and meta-analysis (5). The associations between maternal urinary iodine concentration and SGA versus low birth weight are different. This observed discrepancy can be explained by fundamental differences in how these two outcomes are defined and analyzed. SGA is a relative measure adjusted for gestational age and sex, making it particularly sensitive to detecting abnormalities at the extreme lower end of the growth distribution. In contrast, low birth weight represents an absolute measure where effects can be diluted across the entire birth weight spectrum. Iodine deficiency likely exerts a non-linear, threshold effect—predominantly impacting the most vulnerable infants who fall into the lowest growth percentiles (manifested as SGA), rather than causing a uniform reduction in birth weight across the entire population.

In our study, maternal age was identified as a factor influencing the occurrence of SGA, consistent with a previous cohort study that identified advanced maternal age as a risk factor for SGA (30). With regard to maternal iodine intake, no evidence was found linking the type of salt consumed, the frequency of iodine-rich food intake, or the use of iodine-containing supplements to adverse pregnancy outcomes. The association between maternal iodine intake and adverse pregnancy outcomes has been inconsistently reported across studies (31). Given these discrepancies, further investigation is warranted to clarify the association between maternal iodine status and neonatal outcomes.

In terms of long-term trends in offspring growth, the present study found that maternal iodine insufficiency in the last trimester was associated with lower birth weight and length, as well as reduced long-term length. However, no significant differences were found in offspring weight and length among those with maternal iodine insufficiency in the second trimester. Additionally, maternal iodine insufficiency in the first trimester was linked to lower long-term weight and length; however, no significant associations were identified between maternal iodine insufficiency in the first trimester and birth weight and length. Previous studies have demonstrated that iodine nutritional status during pregnancy in healthy women is associated with offspring growth parameters such as birth weight, birth weight centile, or fetal growth (5, 28, 32, 33). However, the association between maternal urinary iodine concentration (UIC) and offspring birth weight remains inconclusive in the existing literature. A systematic review and meta-analysis demonstrated no significant correlations between maternal UIC during pregnancy and neonatal anthropometric measures (birth weight, length, and head circumference) (34). Therefore, the relationship between maternal iodine status and offspring birth weight remains controversial, warranting further research to clarify this association. Notably, these studies did not assess the long-term effects of maternal iodine deficiency on fetal weight or length. The present findings suggest that iodine status in late pregnancy exerts a more pronounced influence on offspring growth and development. Furthermore, iodine deficiency appears to be more severe in the last trimester. Therefore, future interventions should prioritize evidence-based iodine supplementation and targeted health education for pregnant women in late pregnancy.

This study has several limitations. First, the use of WHO-recommended urinary iodine concentration as an indicator of iodine nutritional status in pregnant women has inherent constraints (16). UIC only reflects recent iodine intake and can be affected by fluid consumption and dietary habits (35, 36). Although 24-hour urine collections can minimize the influence of spot urine concentration variability, they are difficult to obtain in practice (5). Moreover, there is currently no clear diagnostic criterion for serum iodine concentration, which more accurately reflects individual iodine nutritional status (37, 38). To mitigate these limitations, we collected two or more urine samples across different trimesters and categorized UIC into three groups rather than treating it as a continuous variable, thereby reducing the risk of misclassification bias. Second, constraints in the available data also impose limitations on the findings. On one hand, this study only measured urinary iodine levels in pregnant women without assessing maternal and neonatal thyroid hormone profiles, which may introduce bias in the evaluation of thyroid function during pregnancy and in offspring. Future research will enhance the assessment of iodine nutritional status in both pregnant women and their offspring. On the other hand, the study did not account for the impact of neonatal comorbidities on infant weight and length. Infant growth is influenced by multiple factors, including maternal nutritional status, health conditions (such as comorbidities), feeding practices, and complementary feeding. In subsequent studies, we will further investigate the effects of other influencing factors on offspring growth and development, thereby establishing a more comprehensive repository of health influencing factors spanning from the fetal period to infancy. Additionally, participant attrition during offspring follow-up represents another limitation. As some infants underwent pediatric physical examinations at other hospitals, only a subset was assessed at each time point (e.g., 1, 6, and 18 months). To address missing data, the generalized estimating equations model was applied to analyze longitudinal measures. Finally, the relatively small sample size in this study may have limited the statistical power to detect smaller clinical differences, necessitating future validation through larger-scale studies.

Despite these limitations, the present study possesses several strengths. First, iodine nutritional status was assessed separately across different gestational periods, providing insights into the changes throughout pregnancy. UIC in the last trimester was particularly valuable for identifying subclinical iodine deficiency during pregnancy compared with measurements taken in the first and second trimesters. Second, the long-term effects of maternal iodine nutritional status on offspring growth and developmental trajectories were monitored. This information provides essential evidence for guiding appropriate iodine supplementation during pregnancy. These insights hold substantial public health implications.

## 5 Conclusions

In conclusion, the implementation of the new salt iodization strategy in Hangzhou has resulted in insufficient iodine nutritional status among pregnant women, particularly in the second and third trimesters. Consequently, certain pregnant women in Hangzhou, China, remain at risk of subclinical iodine deficiency. Improving urinary iodine levels in this population is essential, as adequate iodine status has been associated with a reduced risk of adverse birth outcomes, such as spontaneous premature birth and SGA infants, while also promoting long-term infant growth. Strengthening the ongoing monitoring of iodine status in pregnant women and implementing targeted measures to improve iodine nutrition—especially in the late stages of pregnancy—are therefore imperative. Furthermore, comprehensive follow-up studies evaluating maternal thyroid function and infant cognitive development are warranted to fully elucidate the long-term effects of maternal iodine deficiency on infant development.

## Data Availability

The original contributions presented in the study are included in the article/supplementary material. Further inquiries can be directed to the corresponding author.
